# AI-Assisted Evaluation of Colon Cleanliness in Capsule Endoscopy Videos

**DOI:** 10.3390/diagnostics15172228

**Published:** 2025-09-03

**Authors:** Pere Gilabert, Carolina Malagelada, Hagen Wenzek, Angus Watson, Alexander R. Robertson, Ádám Finta, Jordi Vitrià, Santi Seguí

**Affiliations:** 1 Departament de Matemàtiques i Informàtica, Universitat de Barcelona, 08007 Barcelona, Spain; 2 Digestive System Research Unit, University Hospital Vall d’Hebrón, 08035 Barcelona, Spain; 3 Department of Medicine, Universitat Autònoma de Barcelona, 08193 Barcelona, Spain; 4 Centro de Investigación Biomédica en Red de Enfermedades Hepáticas y Digestivas (Ciberehd), 08036 Barcelona, Spain; 5 CorporateHealth International, Inverness IV2 5NA, UK; 6 Department of Colorectal Surgery, Raigmore Hospital, NHS Highland, Inverness AB24 3FX, UK; 7 Department of Digestive Diseases, University Hospitals of Leicester NHS Trust, Leicester LE1 5WW, UK; 8 Endo-Kapszula Health Centre and Endoscopy Unit, Department of Gastroenterology, 8000 Székesfehérvár, Hungary

**Keywords:** capsule endoscopy, intraluminal content, minimal annotation, artificial intelligence

## Abstract

(1) **Background**: Accurate evaluation of colon capsule endoscopy videos plays a pivotal role in diagnosing gastrointestinal disorders. A primary step in this process is assessing the cleanliness of the area of interest to determine its admissibility. This study introduces a system designed to assist physicians in evaluating the colon cleanliness score of capsule endoscopy videos. (2) **Methods**: The system uses a TransUNet architecture, a customized loss function, and a low-effort labeling method to propose cleanliness scores for previously unseen videos. The proposed model was evaluated on a dataset of 52 capsule endoscopy videos. Agreement with physicians was measured using Cohen’s kappa statistic. (3) **Results**: The system achieved a Cohen’s kappa agreement of 0.586 with physicians, which is notably higher than the intra-observer variability observed, measured at 0.546. Additionally, the system can show the cleanliness evolution throughout the entire video, helping justify the proposed score. (4) **Conclusions**: The proposed system demonstrates improved agreement with physicians compared to inter-physician agreement, showing its potential to support the cleanliness evaluation process in colon capsule endoscopy. The ability to visualize the cleanliness evolution across the video enhances the transparency and interpretability of the suggested score.

## 1. Introduction

Capsule endoscopy is a minimally invasive diagnostic procedure used to visualize and examine the gastrointestinal (GI) tract. It involves swallowing a small, pill-sized capsule equipped with one or two cameras, a light source, and a transmitter. As the capsule traverses the digestive system, it captures images of the esophagus, stomach, small intestine, and colon. These images are stored in the camera or transmitted wirelessly to a patient’s data recorder, which is later analyzed by medical professionals.

This study focuses on PillCam™ COLON 2, an advanced version of the original COLON 1 capsule. Notably, COLON 2 includes adaptive frame rate (AFR) functionality, which increases image capture speed during faster movement to improve mucosal visualization and battery efficiency. It features dual cameras and wide-angle lenses to enhance coverage. Physicians typically review over 50,000 frames per video using dedicated software, a process that can take up to 90 min per case. While recent software developments have improved navigation and visualization, assessing bowel cleanliness remains subjective and time-consuming, motivating the development of automated evaluation systems like the one proposed in this study.

Capsule endoscopy is particularly beneficial in multiple aspects: It is well tolerated by patients [1] and has the potential to capture the entire GI tract. Moreover, it is well suited as a prescreening technique when a patient needs a quick examination to exclude significant pathology [2]. It allows noninvasive exploration of the colon, aiding in the detection and evaluation of various GI disorders, such as bleeding, ulcers, tumors, and inflammatory bowel diseases.

Performing a colon capsule endoscopy (CCE) involves several important steps to ensure a successful examination. The process begins with bowel preparation, which is crucial for optimal visualization of the GI tract. Bowel preparation typically involves a liquid diet before the procedure and an 8-h fast [3]. In addition, patients may be required to take other medications, such as purgative agents, most commonly Polyethylene Glycol (PEG) [4,5,6], to cleanse the intestines and remove any residual debris or stool.

Despite this preparation, some individuals may not effectively purge their GI tract, leading to the presence of residual intraluminal content. This residue can limit visibility within the intestines, concealing potentially significant findings which may be overlooked by physicians reviewing the procedure. Videos from such procedures become ineffective for patient diagnosis. Consequently, a repeat procedure often becomes necessary, which involves adjustments in bowel preparation to improve cleansing or a switch to a different diagnostic test such as colonoscopy or CT colonography.

An accurate assessment of the cleanliness score immediately after the procedure can accelerate this process. Establishing the level of cleanliness of these videos enables the prompt identification of procedures that require repeating, thus alleviating the workload of physicians and ensuring a more efficient diagnostic workflow. In this work, we propose an Artificial Intelligence (AI)-based system to assist physicians in this initial evaluation step.

Training an AI model usually requires a substantial number of data to achieve the desired results. Obtaining and curating these data is a process that can be quite laborious, demanding significant time and dedication to build a solid training database. Specifically in the context of image segmentation, there is an added layer of complexity. Beyond merely categorizing individual images, one needs to manually outline the area of interest in each frame, making the overall process slower. In our case, if the aim is to create an AI model that computes the visibility of an image, there are two potential approaches: treating it as a classification task, where each image is mapped to a predefined scale of cleansing, or treating it as a segmentation task, predicting the visible mucosa area within each frame.

In this study, we aim to combine the strengths of the two preceding approaches, to reduce the labeling effort while achieving good performance. We train the model to predict segmentation masks that highlight obscured areas within the colon, using only binary labels assigned to randomly extracted image patches. Additionally, we generate a comprehensive summary plot illustrating the visibility across the entire video and categorize it as admissible or not admissible based on the CC-Clear Score.

The field of AI in capsule endoscopy has seen significant advancements in recent years [7]. Various methodologies have been proposed to help in clinical practice, including for landmark localization [8] and detection of multiple pathologies such as Crohn’s disease [9], bleeding [10], polyps [11], or cancer [12]. Efforts have been made to standardize the review process for capsule endoscopy videos [13], but consensus on the reproducibility of the readings remains elusive [14,15,16].

A thorough and safe evaluation of capsule endoscopy videos requires clean visualization of the GI tract [2]. Residual debris can obscure crucial pathology or lesions, complicating the task of reviewing the video and risking missing findings. Assessing how clean a capsule endoscopy video is can be difficult, as it involves looking at both the overall video quality and individual frames. This makes it even harder to agree on a standard way to score them.

Several scales have been proposed to standardize capsule endoscopy readings over the years. The CAC Score was introduced in 2018 [17] and quantifies cleanliness by calculating the percentage of red over green in each frame. The KODA Score [18] uses a two-scale system, evaluating the percentage of visible mucosa and obstructed view, using predefined scores ranging from 0 to 3. Simplifying this approach, the CC-Clear Score [19] assigns cleanliness scores also from 0 to 3 based on thresholds of visible colonic mucosa percentages and was later adapted into the SB-Clear Score [20] to assess cleanliness for the small bowel. The CC-Clear Score has a higher degree of consensus in practice [21] compared to the previous scales and so was selected for our evaluation.

In the AI domain, TransUNet [22] stands out as a pioneering architecture in the field of medical image segmentation [23], combining the strengths of convolutional neural networks (CNNs) and transformers. This hybrid model leverages the robust feature extraction capabilities of CNNs and the powerful global context understanding provided by transformers, which are known for their exceptional performance in natural language processing tasks [24]. Specifically designed for 2D medical images, TransUNet employs a U-Net-like encoder–decoder structure, integrating Vision Transformer blocks [25] within the encoder. This architecture has shown significant improvements in tasks such as organ and tumor segmentation in medical imaging [26], demonstrating superior performance over traditional CNN-based models by effectively addressing the limitations of local receptive fields and capturing more comprehensive contextual information.

Various solutions have been presented involving assessing the cleanliness of capsule endoscopy videos using various models and methodologies. Early approaches, such as those of Buijs et al. [27], utilized support vector machines to classify images into binary categories of dirty and clean. Progressing into the era of deep learning, subsequent works such as that of Noorda et al. [28] employed neural networks to classify patches 64 × 64 pixels in size into these same categories.

More recent efforts, such as those of Nam et al. [29,30], explored systems that classify images into five categories and compared them with cleanliness scores assigned by physicians. Mascarenhas et al. [31] introduced a system to classify images into three categories: excellent, satisfactory, and unsatisfactory. All of these previous works attempted to assign scores to capsule endoscopy videos by classifying either images or patches of images. Ju et al. [32] introduced a system for automatically segmenting intraluminal content and dark areas in images. While the method is interesting, it has a significant drawback: the cost of annotation. It takes a considerable amount of time to effectively segment the GI content in a set of images.

As far as we know, our system is the first to assign CC-Clear Scores to video segments in a way that aligns with physician consensus while requiring minimal annotation effort. Whereas previous methods typically evaluate individual frames and require extensive manual annotations, our system processes entire video segments using weak labels, significantly reducing annotation effort.

## 2. Methods

### 2.1. Methodology

**Figure 1:**
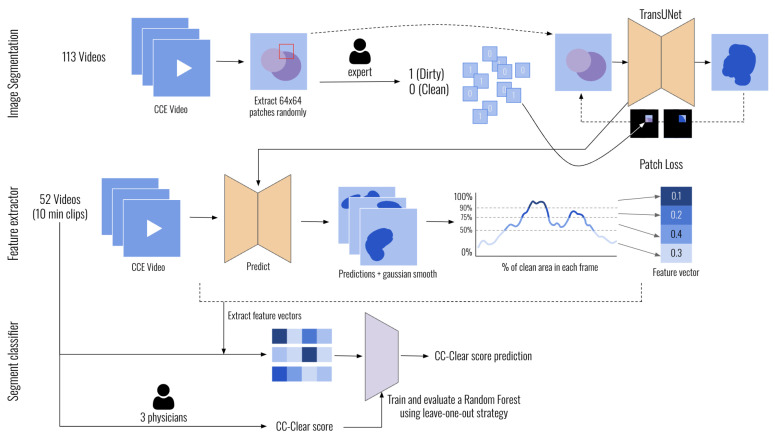
Overview of the method.

The method we present consists of three steps. Figure 1 shows a visual representation of all of them.

1. **Image segmentation**. We train a TransUNet neural network to segment images using patch labels instead of fully annotated segmentation masks. For this, we implemented a custom loss function, which we call “Patch Loss”.
2. **Feature extractor**. Using the predicted segmentation mask for intestinal content in each image, we extract features to assess the cleanliness level of the video.
3. **Segment classification**. Using the features extracted for each video, we predict the CC-Clear Score by training a Random Forest classifier. The scores provided by three expert physicians serve as the ground truth for training.

Our system operates in two main stages: First, we apply a TransUNet-based segmentation model to identify and mask areas of intraluminal content within each frame. Second, we extract features from these masks, which are used to classify video segments according to the CC-Clear scale using a Random Forest classifier. This two-step structure allows us to combine fine-grained frame-level analysis with segment-level cleanliness scoring, mimicking the physician review process more holistically. In the following sections, we explain the full method in further detail.

### 2.2. Image Segmentation

Creating an effective segmentation model typically requires labeled masks for regions containing intraluminal content to serve as the ground truth. These segmentation masks are images of the same size as the originals, highlighting areas obscured by intraluminal content that the model learns to generate. However, this traditional labeling process is both slow and costly.

To optimize the annotation procedure, we propose redefining image labeling by implementing a binary classification task for image patches. In this approach, a label of 1 indicates the presence of intraluminal content that obscures part of the image, potentially concealing a pathology, while a label of 0 signifies the absence of such content. The labeling criteria are straightforward: a patch receives a label of 1 if a physician determines that the intraluminal content in the patch could hide a pathology, and 0 otherwise.

Traditionally, classifying clean and dirty patches has been carried out using only the patches themselves, without considering the entire image context [28]. We propose maintaining this simple approach for patch annotation but leveraging this information within the loss function during segmentation model training. By incorporating patch-level labels into the loss function, we can condition the segmentation model on the detailed patch information we have.

To implement this, we use TransUNet, which takes an input of (256,256,3) and produces a corresponding mask of the same size. For a given image $X_{k}$ and a patch $P_{k}$ within that image, we define the *Patch Loss* as a cross-entropy loss restricted to each patch. For a batch of *B* patches, this loss function can be expressed as follows:

$$\begin{array}{cc} \; & \mathrm{Patch}\;\mathrm{Loss}=-\frac{1}{B}\sum_{k=1}^{B}L_{k}\\ \; & L_{k}=\sum_{i=1}^{H}\sum_{j=1}^{W}\left(y_{k}\log\left(P\left(X_{k}^{\left(i,j\right)}{|}_{P_{k}},y_{k}\right)\right)+\left(1-y_{k}\right)\log\left(1-P\left(X_{k}^{\left(i,j\right)}{|}_{P_{k}},y_{k}\right)\right)\right) \end{array}$$

where $X_{k}^{\left(i,j\right)}$ represents the pixel at coordinates (i,j), $y_{k}$ is the binary label assigned to the patch by the expert, and H,W are the height and width of the patch, respectively. In our setup, both dimensions *H* and *W* are set to 64 pixels.

To smooth the result, we apply a Gaussian kernel of size 0.4. The final segmentation masks are generated by classifying pixels with an activation higher than 0.5 as part of the segmented region, resulting in binary masks for each frame.

### 2.3. Feature Extractor

Using the segmentation masks from the previous step, we calculate the area covered by the segmentation. Specifically, we measure the proportion of pixels in the segmentation mask relative to the total number of pixels in the image, excluding the black areas in each corner.

With these per-image scores, we generate a plot showing the visibility score for each frame throughout the entire video segment. This plot visualizes the evolution of visibility across the segment and highlights regions where the capsule is stuck in zones of poor visibility.

**Figure 2:**
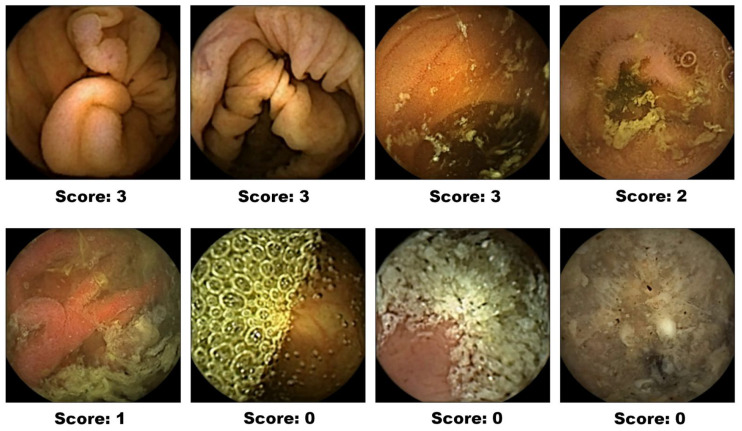
CC-Clear Score examples.

From this visibility plot, we extract features aligned with the CC-Clear Score. We categorize and count the number of frames based on their visibility levels: less than 50%, between 50% and 75%, between 75% and 90%, and greater than 90%. These four visibility metrics are then used in the final step of our analysis. Representative images for each CC-Clear Score category (0–3) are illustrated in Figure 2, providing visual guidance for interpreting these visibility levels.

### 2.4. Segment Classification

Achieving reproducibility in assigning cleanliness scores to entire videos is challenging due to significant inter-interpreter variability [14]. To address this, we propose validating our system at the segment level by analyzing 10-minute clips. This approach allows for a more manageable and consistent assessment of video cleanliness.

We randomly selected 10-minute clips (considering actual capsule movement time) from the videos. These clips were independently evaluated by three experienced physicians. Each physician assigned a CC-Clear Score ranging from 0 to 3, reflecting the cleanliness level of each segment.

Using the features extracted in the previous steps, we trained a Random Forest classifier with a leave-one-out cross-validation strategy [33]. The physicians’ scores served as the ground truth for training. We explored two approaches:

- **Individual Model Training**: We trained separate models to replicate the scoring patterns of each physician.
- **Consensus Model Training**: We trained a single model using the consensus score derived from the three physicians as the ground truth. This consensus score was calculated by averaging the individual scores given by the physicians and rounding the result to the nearest integer.

This dual approach allowed us to capture both individual scoring nuances and a consensus view of video cleanliness. For each method, we assessed the agreement between the physicians’ scores and the model’s predictions.

### 2.5. Dataset

This study utilizes 165 CCE videos sourced from two retrospective studies conducted at the facilities of the NHS Highlands Raigmore Hospital in Inverness. Both studies included patients who were referred with symptoms or for surveillance within the Highlands and Islands area of Scotland. All these patients had a positive Fecal Immunochemical Test (FIT) result.

All patients followed the ScotCap (NHS Scotland) bowel preparation protocol [34]. Three days before the procedure, participants began with Macrogol 3350 twice daily, transitioning to a low-residue diet two days prior. A clear liquid diet was observed the day before the procedure. PEG bowel preparation involved 2L the evening before and 2L the morning of the exam, each consumed over approximately 2 h.

The videos were captured using a PillCam^TM^ COLON 2, which contains two cameras (front and rear). To ensure patient confidentiality, all videos were anonymized, removing relevant information stamped on the images.

The capsule endoscopy videos were independently reviewed by three physicians (MDs) from Germany and the United Kingdom (2 male, 1 female) with case experience ranging from 500 to 3000 CCE procedures. While no formal certification system exists for capsule endoscopy in these countries, all physicians adhered to the European Society of Gastrointestinal Endoscopy (ESGE) guidelines and completed both an internal training protocol and the Medtronic online certification program.

### 2.6. Data Splits

To ensure fair training, evaluation, and testing, we split the videos into two main groups to avoid data leakage between the steps. The total set of 165 videos was split in the following way:

- We used 113 videos to train, validate, and test the image segmentation model. From these videos, a total of 8492 patches, each of size 64 × 64 pixels, were randomly extracted. The dataset of 113 patients was divided into three groups, 69 patients for training (5306 patches), 22 patients for validation (1539 patches), and 22 patients for testing (1647 patches), to evaluate the model’s performance.
- The remaining 52 videos were used to train and evaluate the performance of the segment classifier using a leave-one-out strategy.

### 2.7. Training Configuration

All the deep learning code was implemented in Python and executed on an NVIDIA 3090 RTX GPU.

We used Keras as a framework to reproduce the strategy of Noorda et al. [28] and we integrated a pre-trained TransUNet model as the core component of our approach.

To evaluate the effectiveness of our solution, we not only performed the segmentation task but also compared our method against other classifiers for patch classification. Although our primary focus was on segmentation rather than classification, we assigned labels to patches based on the model’s segmentation output. Specifically, a patch was labeled as positive (Dirty, 1) if the predicted segmented area covered 50% or more of the patch. Conversely, it was labeled as negative (Clean, 0) if less than 50% of the patch was segmented.

For the CC-Clear Score classifier, we employed a leave-one-out cross-validation strategy using the remaining 52 videos. This approach involved training a model on a subset of 51 videos and testing it on the 1 remaining video, ensuring that every video was used for testing exactly once. We trained a Random Forest classifier with 100 estimators and set the maximum depth to 2 to prevent overfitting. Standard algorithms from the sklearn library were utilized throughout the process.

## 3. Results

The results we present are organized in the following way: Firstly, we present an evaluation of the image segmentation. Secondly, we compare the patch classification performance. Lastly, we present results on the segment classification.

### 3.1. Segmentation Results

We initially demonstrated the model’s ability to segment intraluminal content by requesting an expert to manually segment a small random set of 32 images. Table 1 presents the mean Intersection over Union (mIoU) results comparing the predicted masks to the ground truth. Our model, shown in the final row, exhibits improved segmentation masks with the introduction of this conditioned loss.

**Figure 3:**
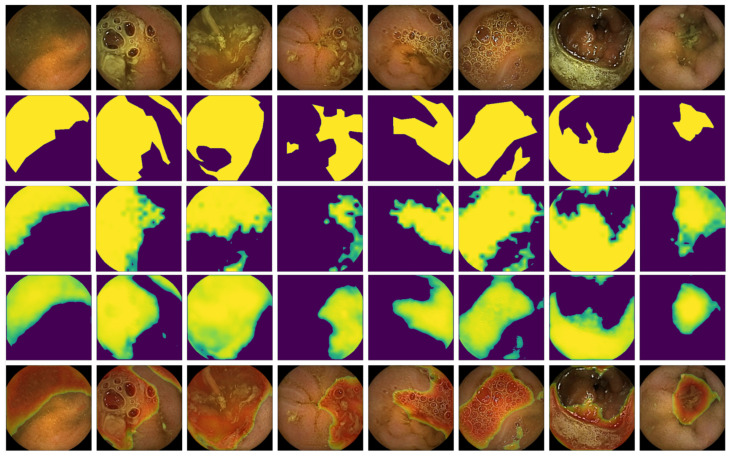
Segmentation results using the TransUNet + Patch Loss strategy on randomly selected images annotated by an expert. Rows are ordered as follows: original image, expert-annotated ground truth mask, predicted mask from the Noorda et al. [28] model, predicted mask from our proposed model, and the thresholded mask from our model using a 0.5 cutoff.

Figure 3 showcases the segmentation performance of the TransUNet with the Patch Loss strategy. Each row in the figure sequentially displays the original images, the ground truth masks manually annotated by an expert, the masks predicted by the Noorda et al. [28] model, the masks predicted by our model, and the final binary masks obtained by applying a 0.5 threshold to our model’s predictions. This visual comparison illustrates the accuracy and effectiveness of our approach in segmenting intraluminal content with a low-annotation strategy. It is important to highlight the inherent difficulty in segmenting intraluminal content accurately. As evident in the images, the boundaries of the areas occluded are often not clearly defined. This lack of clear demarcation makes segmentation particularly challenging and leads to significant variability in the masks depending on the expertise and interpretation of the annotator.

### 3.2. Patch Classification Results

We evaluated patch classification using the method previously explained to compare our proposed strategy with other baseline methods.

We selected ViT-B16 and ResNet50 as baseline models for comparison due to their prominence in image analysis. ViT-B16 represents a pure transformer-based model, offering a relevant benchmark for our hybrid TransUNet approach. ResNet50, a standard CNN architecture, is widely used in medical imaging and serves as a robust convolutional baseline. Additionally, we reproduced the method proposed by Noorda et al. [28], which is a domain-specific baseline developed for capsule endoscopy patch classification. Table 2 compares these methods against our proposed TransUNet + Patch Loss, evaluated on the test set of 22 videos. The results clearly demonstrate that our proposed strategy surpasses the previous methods across all metrics presented.

### 3.3. Segment Classification

We randomly extracted 10-minute clips from 52 new videos, one per video, and three expert physicians evaluated their cleansing. Following the CC-Clear Score, each clip received a score between 0 and 3, with 0 being a video almost without visible mucosa, and therefore unusable, and 3 a video with a very clean mucosa without any doubt of missed pathology because of the presence of intraluminal content. The scores that the physicians reported are shown in Table 3. A moderate agreement based on the Cohen’s kappa score was found between the experts: $k_{12}=0.537$, $k_{23}=0.459$, and $k_{13}=0.643$, where $k_{ij}$ represents the score between physician *i* and physician *j*. The average score among the physicians was ${\bar{k}}_{\mathrm{orig}}=\frac{1}{3}\left(k_{12}+k_{23}+k_{13}\right)=0.546$. These results highlight the significant inter-observer variability previously noted in the existing literature.

**Figure 4:**
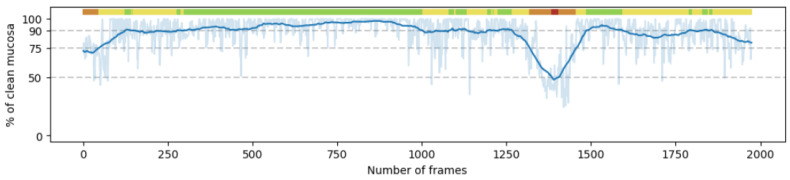
Example of a test procedure. Clean mucosa prediction for each frame in the clip. A centered moving average is applied to smooth the results. At the top of the plot, the predicted CC-Clear Score for each part of the video clip is shown using a color scale, Red (<50%), Orange (50–75%), Yellow (75–90%), and Green (>90%), which matches with the thresholds set by the horizontal dashed lines.

Figure 4 shows a test procedure with the predicted cleanliness of each frame. Horizontal dashed lines show the different thresholds set for the CC-Clear Score. These thresholds are better visualized in the colored bar at the top of the plot, summarizing the cleanliness score of each area of the video. Depending on the level of cleanliness of the neighboring frames, the scale is Red (<50%), Orange (50–75%), Yellow (75–90%), or Green (>90%). The plot shows the predicted percentage of clean mucosa and a centered moving average for better visualization.

**Figure 5:**
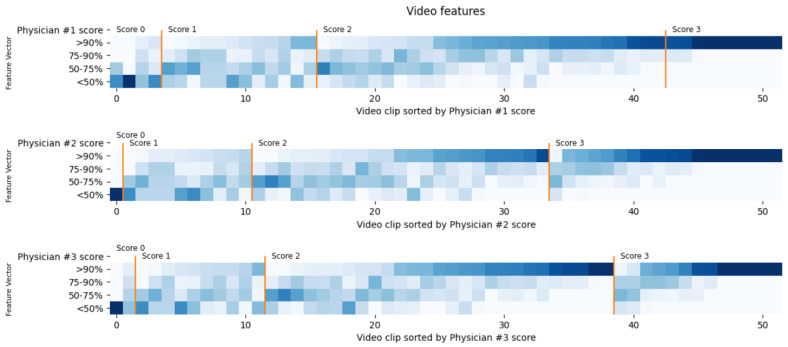
Feature vector for each video. Each video is a column showing its 4 values. A darker color means a higher value. Values are sorted first by ground truth (physician score) and then by the first component of the vector (first row of each plot).

For each clip, we extracted four features based on the number of frames in each of the four regions of visibility, obtaining a four-dimensional vector representing the video. Figure 5 shows a visual correlation between the features and the ground truth established by the physicians. We can observe that, while the extremes seem more homogeneous, the central area of the plots is more ambiguous.

Following the process explained in the methodology, we evaluated the system using two strategies: Individual Model Training and Consensus Model Training.

#### 3.3.1. Individual Model Training

**Figure 6:**
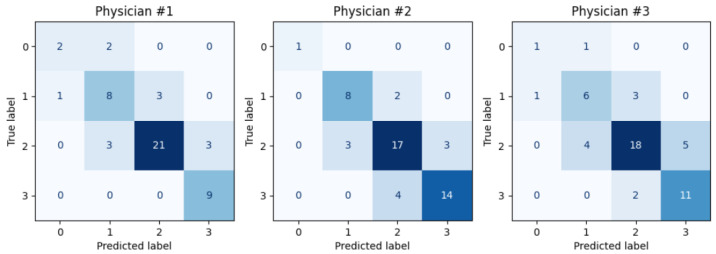
Confusion matrices for the three models. Each model is trained on the scores of a single physician using a leave-one-out strategy.

We independently trained a model to replicate the scoring of each of the physicians. Figure 6 presents the confusion matrices for these three models. The first model, trained to mimic the first physician, achieves a Cohen’s kappa agreement of $k_{1}=0.649$ with an accuracy of 76.9%. The second model, aligned with the second physician, reaches an agreement of $k_{2}=0.645$ and also an accuracy of 76.9%. The third model, corresponding to the third physician, attains an agreement of $k_{3}=0.528$ with an accuracy of 69.2%. The average agreement across these individual models is ${\bar{k}}_{\mathrm{indiv}}=0.607$. Notably, both the mean agreement and each individual model’s agreement with their respective physician exceed the average original inter-observer agreement between the physicians, which was ${\bar{k}}_{\mathrm{orig}}=0.546$.

#### 3.3.2. Consensus Model Training

**Figure 7:**
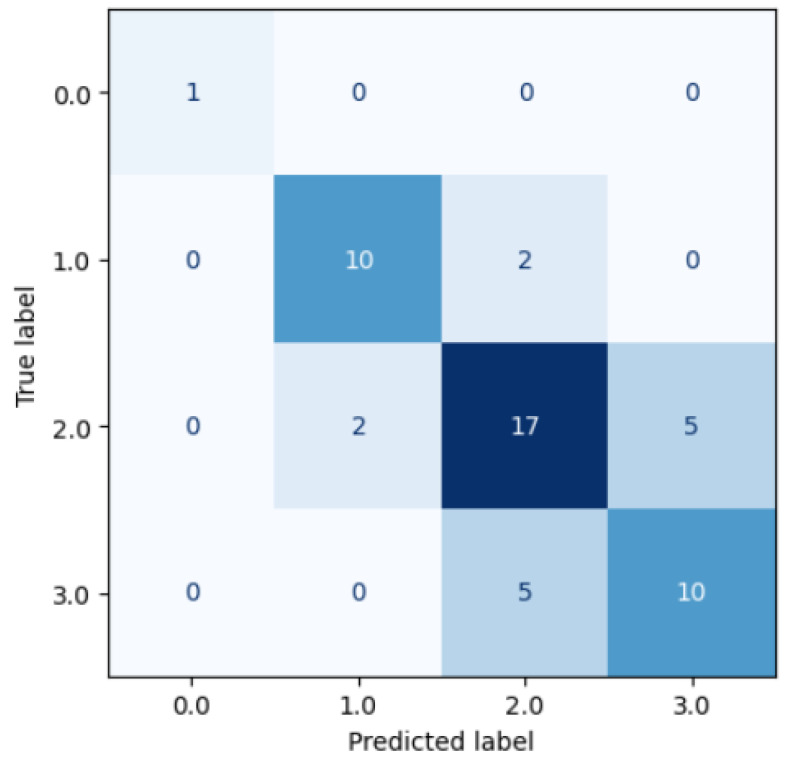
Results of a regressor model trained using the average of the physicians’ scores as the ground truth.

We also developed a Random Forest classifier model using the average of the physicians’ scores, rounded to the nearest integer, as the consensus ground truth. This approach simulated the combined consensus scoring of each video by the three physicians. Figure 7 shows the confusion matrix for the consensus model’s results.

The consensus model achieved a Cohen’s kappa agreement of $k_{\mathrm{cons}}=0.586$, which is an improvement over the average original inter-observer agreement between the experts, ${\bar{k}}_{\mathrm{orig}}=0.546$.

The Random Forest was selected as it was the best performer among the classifiers evaluated. Table 4 shows the agreement scores, ${\bar{k}}_{\mathrm{indiv}}$ and $k_{\mathrm{cons}}$, resulting from the application of different classification methods from the scikit-learn library.

Table 5 summarizes all the numbers mentioned in the previous sections.

## 4. Discussion

We introduce a novel method aimed at improving the classification and segmentation of intraluminal content with minimal labeling effort. By leveraging our model’s training approach, we observed improvements in segmentation mask accuracy compared to other low-effort annotation methods. Our process involves several stages, each validated with different CCE videos to ensure robustness and prevent data leakage.

To support our claims, we conducted a series of experiments. Initially, we evaluated the segmentation performance without relying on fully annotated masks. We achieved a notable mIoU score by introducing a conditional loss function on annotated patches. Subsequently, we assessed patch classification accuracy across all models, with our newly proposed model performing with higher accuracy.

Given the significant variability in determining cleanliness scores for entire videos, as noted in the literature, we focused on evaluating our method using 10-minute clips from various CCE videos. Three physicians provided cleanliness scores on a scale from 0 to 3 for each clip. Using these scores, we trained Random Forest classifiers—one for each physician—to replicate their scoring patterns. The models exhibited stronger agreement in replicating physician assessments than the individual scores provided by the physicians. Specifically, the mean achieved agreement of the models was ${\bar{k}}_{\mathrm{indiv}}=0.607$, surpassing the original inter-physician agreement, which was ${\bar{k}}_{\mathrm{orig}}=0.546$.

Furthermore, we developed a jointly trained Random Forest classifier model using the physicians’ average scores as consensual ground truth. This model, which simulates a consensus score by three physicians, achieved an agreement of $k_{\mathrm{cons}}=0.586$, also an improvement when compared to the original inter-physician agreement.

Despite the promising results, several factors can influence the system’s accuracy. One major limitation arises from the ill-defined boundaries of intraluminal content, which complicate the segmentation task and introduce variability in both manual and model-generated masks. This variability is particularly pronounced in intermediate CC-Clear Scores, where partial visibility leads to greater subjectivity, as illustrated in Figure 5 and Figure 6. While the model performs well on clearly clean or clearly dirty segments, its performance may degrade in ambiguous cases. These challenges reflect real-world variability in human interpretation ($k_{\mathrm{cons}}=0.586$), highlighting the need for continued refinement, especially in borderline cases.

Our approach shows strong potential for cost-effectiveness in clinical settings. By achieving high segmentation and classification performance using only sparsely annotated data, the method reduces the time and resources required for manual labeling. Automated cleanliness scoring can alleviate physician burden by streamlining review and improving consistency, particularly in large-scale screening programs. More reliable assessment may also decrease the need for repeat procedures due to inadequate preparation, enhancing clinical efficiency and patient experience. While results are encouraging, further validation in larger clinical studies is needed to confirm generalizability. Nonetheless, our physician-informed consensus model offers a promising step toward scalable, accurate, and efficient intraluminal content analysis in capsule endoscopy.

## Figures and Tables

**Table 1:** Mean Intersection over Union score evaluated on 32 images manually segmented by an expert annotator.

Strategy	mIoU
Noorda et al. [28]	0.43
ResNet50	0.48
ViT-B16	0.55
**TransUNet + Patch Loss (Ours)**	**0.73**

**Table 2:** Results of the four strategies evaluated on the test set. Results show that the proposed strategy, TransUNet + Patch Loss, improves patch classification.

Strategy	Acc.	AUC	Prec.	Rec.	F1
Noorda et al. [28]	0.89	0.82	0.92	0.67	0.78
ResNet50	0.89	0.87	0.75	0.84	0.79
ViT-B16	0.90	0.88	0.84	0.82	0.83
**TransUNet + Patch Loss (Ours)**	**0.97**	**0.96**	**0.93**	**0.93**	**0.93**

**Table 3:** Video clip scores. Number of videos each physician scored for each different score.

CC-Clear Score	0	1	2	3	Mean Score
**Physician #1**	4	12	27	9	1.79 ± 0.82
**Physician #2**	1	10	23	18	2.12 ± 0.78
**Physician #3**	2	10	27	13	1.98 ± 0.78

**Table 4:** Results of the individually trained models and the consensus-trained model with different classification algorithms. All of them use the same four features for classification.

Method	${\bar{k}}_{\mathrm{indiv}}$	$k_{\mathrm{cons}}$
Logistic Regression	0.370	0.245
K-Nearest Neighbors (3 neighbors)	0.545	0.461
SVM (linear kernel)	0.334	0.282
SVM (rbf kernel)	0.440	0.509
SVM (polynomial kernel, degree 2)	0.451	0.543
**Random Forest**	**0.607**	**0.586**

**Table 5:** Summary of agreement scores: physicians, individual models, and consensus approach.

	Physician			Individual Model			Consensus Model
	$P_{1}$	$P_{2}$	$P_{3}$	$M_{1}$	$M_{2}$	$M_{3}$	$M_{\mathrm{cons}}$
$P_{1}$	-	$k_{12}=0.537$	$k_{13}=0.643$	$k_{1}=0.649$	-	-	$k_{\mathrm{cons}}=0.586$
$P_{2}$	-	-	$k_{23}=0.459$	-	$k_{2}=0.645$	-	
$P_{3}$	-	-	-	-	-	$k_{3}=0.528$	
avg.	${\bar{k}}_{\mathrm{orig}}=0.546$			${\bar{k}}_{\mathrm{indiv}}=0.607$			-

## Data Availability

The datasets used and analyzed in this paper are available from the author upon reasonable request.
